# Using the Neandertal genome to study the evolution of small insertions and deletions in modern humans

**DOI:** 10.1186/s12862-017-1018-8

**Published:** 2017-08-04

**Authors:** Manjusha Chintalapati, Michael Dannemann, Kay Prüfer

**Affiliations:** Max Planck Institute for Evolutionary Anthropology, 04103 Leipzig, Germany

**Keywords:** Neandertal, Ancient DNA, Indel evolution

## Abstract

**Background:**

Small insertions and deletions occur in humans at a lower rate compared to nucleotide changes, but evolve under more constraint than nucleotide changes. While the evolution of insertions and deletions have been investigated using ape outgroups, the now available genome of a Neandertal can shed light on the evolution of indels in more recent times.

**Results:**

We used the Neandertal genome together with several primate outgroup genomes to differentiate between human insertion/deletion changes that likely occurred before the split from Neandertals and those that likely arose later. Changes that pre-date the split from Neandertals show a smaller proportion of deletions than those that occurred later. The presence of a Neandertal-shared allele in Europeans or Asians but the absence in Africans was used to detect putatively introgressed indels in Europeans and Asians. A larger proportion of these variants reside in intergenic regions compared to other modern human variants, and some variants are linked to SNPs that have been associated with traits in modern humans.

**Conclusions:**

Our results are in agreement with earlier results that suggested that deletions evolve under more constraint than insertions. When considering Neandertal introgressed variants, we find some evidence that negative selection affected these variants more than other variants segregating in modern humans. Among introgressed variants we also identify indels that may influence the phenotype of their carriers. In particular an introgressed deletion associated with a decrease in the time to menarche may constitute an example of a former Neandertal-specific trait contributing to modern human phenotypic diversity.

**Electronic supplementary material:**

The online version of this article (doi:10.1186/s12862-017-1018-8) contains supplementary material, which is available to authorized users.

## Background

Recent advances in sequencing technology and laboratory methods made it possible to sequence complete genomes from ancient DNA preserved in human remains [1, 2]. High-coverage genome sequences were recently generated from ancient humans, including those from a Neandertal individual [3], a member of a group of close extinct relatives of all present-day humans. The sequence of the Neandertal genome provides a unique resource to study evolution since it can be used to sort sequence changes on the human lineage into those that likely occurred recently (i.e. those that are not shared with the Neandertal) and those that occurred earlier. Of particular interest are those modern human changes that rose to high frequency or reached fixation since the split from Neandertals, since these changes may underlie phenotypes that were advantageous during the evolution of modern humans. Among the sequence changes reaching fixation are also 4113 insertion/deletion variants [3].

The study of the high-coverage Neandertal genome confirmed that modern humans outside of Africa trace a small percentage of their ancestry back to an admixture event with Neandertals [3]. Although likely of small magnitude, the admixture event occurred sufficiently recent so that a large fraction (around 40%) of the Neandertal genome sequence segregates within present-day humans [4, 5]. However, not all regions in the genome show an equal fraction of Neandertal ancestry, suggesting that a substantial fraction of the introgressed material was lost due to negative selection [4–8], while some specific variants rose to higher frequency likely because they conveyed a selective advantage to the carriers [9–13]. Among the introgressed variants are also larger deletions, some of which are overlapping exons [14].

Although most of the sequence variation among human individuals is due to single nucleotide changes, insertion/deletions (indels), which are approximately one order of magnitude less abundant, have a higher probability to affect function than nucleotide substitutions [15]. However, indels are often excluded in evolutionary studies. This is likely due to the particular challenges of indel genotyping [16–18] and the heterogeneous processes generating indels that lead to a large variation in mutation rates along the genome [19, 20]. For example, deletions were found to evolve, on average, under stronger negative selection on the human lineage than insertions by one study that compared fixed to polymorphic indels [21], while a later study found the opposite signal using the allele frequency spectrum between populations [22]. The cause for this discrepancy may lie in homoplasy, i.e. the independent occurrence of identical changes on several lineages, which can lead to the mis-assignment of the ancestral state and type of the mutation (insertion or deletion) [19].

Here, we use the Neandertal genome [3] together with data of present-day humans from the 1000 Genomes data [23] to identify indels and divide the set of indels further into those that likely occurred after the split from Neandertals, those that arose before the split from Neandertals and likely introgressed indels. We test for different patterns of selection between these sets and compile a list of introgressed and modern-human-fixed indels that may contribute to modern human phenotype.

## Results

### Indels on the human lineage

To identify insertion and deletion events on the modern human lineage and to alleviate the problem of mis-assignment of the ancestral state, we aligned the human reference genome with seven primate genomes and inferred the derived state on the human lineage by requiring an identical ancestral allele in all seven primate genomes. An insertion on the human lineage is called only when all non-human primates show a deletion compared to the human state, and a human-specific deletion when all primates show an insertion. Our method detected 315,513 indels of 1-5 bp in length in the human reference genome. Of these, most indels (315,412) were covered in the high-coverage Neandertal genome [3].

**Fig. 1:**
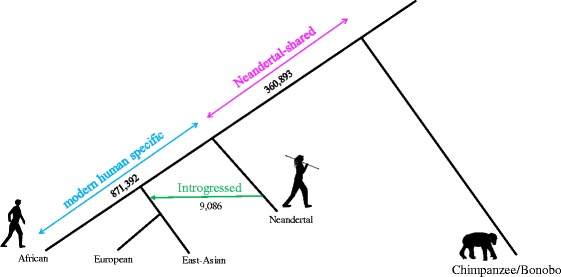
Indels analyzed in this study. Indels on the human lineage divided into three categories: *a*) Indels which likely arose on the human lineage after the split from Neandertals and are specific to modern humans (*blue*) *b*) Indels which occurred before humans split from Neandertals and are shared with Neandertals (*pink*) *c*) Indels introduced into non-Africans due to introgression from Neandertals (*green*)

We used data from the 1000 Genomes project phase 3 [23] to further increase the set of variable indels. Variants marked as copy number variants (“<CN>”) exceeded the length of variants considered here and were excluded. A total of 2,982,740 were inferred from 1000 Genomes data after filtering out sites with more than one derived variant. These indels were assigned an ancestral and derived state by comparison to seven non-human primate genomes, and overlapped with the Neandertal genotypes, resulting in 989,138 indels of length 1-5 bp. Combining indels identified using the human reference and those identified using the 1000 Genomes data, yielded 1,232,285 indels of size 1-5 bps on the human lineage (245,520 appear fixed and 986,765 were segregating in present day populations) (Fig. 1, Additional file 1: Figure S1).

We computed the ratio of deletions to insertions for fixed (1.45) and polymorphic indels (2.06) and found ratios higher than 1, consistent with deletions accumulating approximately twice as fast as insertions [21, 24–26].

### Modified McDonald–Kreitman test on the human lineage indels

Previous studies have used a modified version of the McDonald-Kreitman test [19, 21, 27] -- comparing the ratio of fixed deletions to fixed insertions to the ratio of polymorphic deletions to polymorphic insertions -- to test whether insertions and deletions are affected differently by selection. Under neutrality both the fixed and polymorphic ratios are solely dependent on the rates at which insertions and deletions are generated, i.e. at a roughly 2-fold higher rate for deletions than for insertions. Under this assumption, the ratios of deletions to insertions are not expected to differ significantly from each other when comparing fixed to polymorphic sites. However, a departure from this expectation can emerge if one type of change is selectively favored over the other, and is thus biased towards fixation. Note that such a signal requires only the average selection pressures on insertions and deletions to differ; the majority of both types of changes can still be selectively neutral.

We first applied the modified McDonald Kreitman test to all 1–5 base pair long indels described in the previous section and found a significant difference between the ratio of fixed to the ratio of polymorphic indels (*p* < 2.2e-16). In order to test whether this signal is driven by a certain length of indels, we repeated the test for each length, separately, and found that the signal persists in all comparisons (Table 1). This result is consistent with the results of Kivkstat and Duret [19] and Sjödin et al. [21] suggesting that deletions are under stronger negative selection than insertions.

**Table 1:** Fixed and polymorphic indels on the human lineage by length

Category	1 bp	2 bp	3 bp	4 bp	5 bp	Sum: 1–5 bp
Fixed deletions	86,791	26,860	14,802	12,161	4689	145,303
Fixed insertions	66,333	13,589	8022	9406	2867	100,217
Fixed rDI	1.30	1.97	1.845	1.29	1.635	1.449
Polymorphic deletions	344,533	121,548	82,114	84,393	31,607	664,195
Polymorphic insertions	226,712	38,545	21,147	27,180	8986	322,570
Polymorphic rDI	1.519	3.15	3.88	3.10	3.52	2.06

Ratio of deletions to insertions (rDI) is given for polymorphic and fixed indels of different lengths on the human lineage. Fisher’s exact tests were applied to the counts of fixed and polymorphic insertions and deletions in each column and yielded *p*-values < 2.2e-16 in all comparisons

It is interesting to note, that the ratio of polymorphic insertions and polymorphic deletions also differs significantly between all lengths (pairwise comparisons between lengths 1-5 bps: *p*-values < 0.05).

### Derived allele frequency of the human lineage indels

**Fig. 2:**
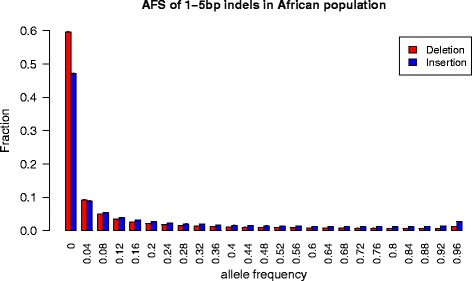
Derived allele frequency spectra (AFS) of indels in Africans from the 1000 Genomes dataset. The AFS for non-African populations is shown in Additional file 1: Figure S1. Wilcoxon rank sum tests (two-sided) show that the frequency distributions of insertions and deletions differ significantly for all populations (*p* < 2.2e-16)

The derived allele frequency spectra (AFS) of polymorphic insertions and deletions can be used as an alternative to test for differences in selection pressure affecting both types of changes [28]. The test is based on the idea that a favorable allele will on average segregate at higher frequency compared to neutral alleles, and neutral alleles will in turn segregate at higher frequencies compared to deleterious alleles [29]. We found that the AFS for deletions differs significantly from the AFS for insertions (two-sided Wilcoxon rank sum test; *p* < 2.2e-16; Fig. 2), with deletions showing an excess of low-frequency alleles compared to insertions. This signal is detected consistently in all 1000 Genomes populations and for all sizes of indels (1-5 bp) (Additional file 1: Figure S2).

### Genomic distribution of the human lineage indels

**Fig. 3:**
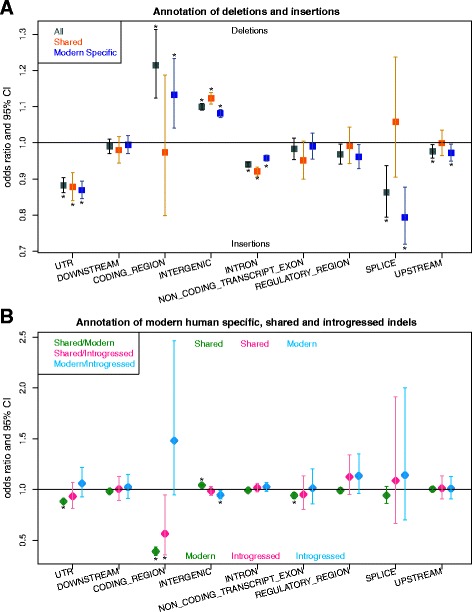
Proportion of different types of indels in classes of genomic regions. Odds ratios with 95% confidence intervals comparing **a** insertions to deletions, and **b** modern human specific, Neandertal-shared and introgressed indels. Categories with FDR adjusted *p* < 0.05 are marked with (*)

The previous two tests examined the difference in selection pressure between insertion and deletions by comparing allele frequencies. However, if one type of change is more often deleterious, a difference may also be visible in the fraction of insertions and deletions residing in regions that are more likely functional as compared to regions that are more likely neutral. We tested this hypothesis by annotating indels by their genomic location using the Variant Effect Predictor [30]. As expected, a major fraction of indels fall in intronic and intergenic regions while a much smaller fraction fall in coding regions. In addition, intergenic regions show a statistically significant higher fraction of deletions than insertions (binomial test; *p* = 7.3e-119; FDR adjusted *p =* 7.8e-117) while the opposite is true for intronic regions (*p*-value = 3.6e-59; FDR adjusted *p* = 1.3e-57; Fig. 3a). This observation is compatible with the notion that deletions are more constraint than insertions. However, we caution that differences in insertion and deletion frequencies may also be influenced by other factors, such as sequence context [31–33] leading to unequal insertion and deletion mutation rates between classes of genomic regions.

### Modern human specific and Neandertal-shared indels

We divided indels into those that were identified in the genomes of the modern human reference and the Neandertal, and those that were only detected in the human reference. A total of 37,443 indels were modern human specific and 265,975 were shared. The frequency of modern human specific indels can be used to calculate a relative divergence of the human reference to the Neandertal genome. We calculate a divergence of 12.3% relative to the divergence to the common ancestor with chimpanzee, close to the range of values calculated using nucleotide differences (11.2–11.8%, see SI6a in [3]).

We classified polymorphic indels from the 1000 Genomes Project [23] into those for which the derived variant is shared with the Neandertal and those where the derived variant is only observed in modern humans, and pooled the dataset with human-reference specific indels. As expected by the difference in age, the majority of the 360,893 shared indels were fixed (243,060 fixed and 117,833 polymorphic) while the majority of the 871,392 modern human specific indels were polymorphic (2460 are fixed and 868,932 are polymorphic).

Neandertal-shared indels are expected to be on average older than indels that are specific to modern humans. We use this expectation to test again for differences between the ratios of deletions to insertions of both age-classes, similar to the McDonald-Kreitman test. The ratio of deletions to insertions is significantly lower for shared compared to modern human specific indels (Table 2, Additional file 1: Table S6A) consistent with earlier comparisons between fixed and polymorphic indels. When annotating indels with the class of genomic regions that is most likely to influence phenotype, we find that a significantly higher fraction of Neandertal-shared indels fall in intergenic regions compared to modern human specific indels (Fisher’s exact test; *p* = 1.77e-21; False Discovery Rate (FDR) adjusted *p* = 9.57e-21; odds ratio: 0.96) while modern human specific indels fall more often in intronic regions compared to shared indels, although this difference is not significant after multiple testing correction (Fisher’s exact test; *p* = 0.04, FDR adjusted *p* = 0.08; odds ratio: 1.009). These signals are consistent with a longer exposure to selection for Neandertal-shared indels as compared to modern human specific indels (Fig. 3b). For both classes, a higher fraction of insertions resides in coding regions compared to deletions and the opposite pattern is observed for intergenic regions (Fig. 3a).

**Table 2:** Contingency table contrasting modern human specific indels and shared indels

Category	Shared	Modern Human specific
Deletions	205,075	604,423
Insertions	155,818	266,969
Ratio(Deletions/Insertions)	1.316	2.26

The ratios of deletions to insertions are significantly different between the shared and modern human specific classes (Fisher’s exact test; *p* < 2.2e-16, odds ratio = 0.58)

### Putatively introgressed indels

A subset of the indel variants segregating in non-African populations trace their ancestry back to Neandertals, through an admixture event between non-Africans and Neandertals 50–60 thousand years ago [34, 35]. By conditioning on the absence of the derived variant in Africans and the presence of the derived variant in Neandertals and either the East-Asian or European population, we identified 9086 putatively introgressed indels. Of these 6070 are deletions and 3016 insertions with an average allele frequency of 0.027 in Europeans and 0.048 in the East-Asian population (Wilcoxon rank test for European frequencies smaller less than East-Asian frequencies: *p* = 1.8e-35). The difference in allele frequencies between both populations is similar to the one observed for putatively introgressed SNPs (Europeans: 0.026; East-Asians: 0.046; Additional file 1: Figure S4). Following the patterns observed for all indels, we found that a higher fraction of introgressed deletions fall in intergenic regions compared to introgressed insertions (Additional file 1: Figure S3). Our previous results, comparing modern human specific to Neandertal-shared indels, remain significant when putatively introgressed indels are removed (Additional file 1: Tables S6A, 6B).

To gain insight into the selection pressures that acted on introgressed indels, we compared their distribution over classes of genomic regions with those of Neandertal-shared (but without introgressed) and modern human specific indels (Fig. 3b). Interestingly, we find that a slightly smaller proportion of introgressed indels fall in intron regions compared with the other two classes of indels (55.3% versus 55.7% and 55.9% for Neandertal-shared and human specific, respectively), and a slightly larger proportion of introgressed indels fall into intergenic regions (31.5% versus 31.2% and 30.3%) (Additional file 1: Table S5). For Neandertal-shared variants this difference to introgressed indels is not statistically significant (Fisher’s exact test, one-sided, *p* = 0.23, odds ratio: 1.016 and *p* = 0.26, odds ratio: 0.985 for intron and intergenic regions, respectively), while modern human specific variants show a significant difference to introgressed variants for intergenic (*p* = 0.007; FDR adjusted *p* = 0.02; odds ratio: 0.945) but not intron regions (*p* = 0.13, odds ratio: 1.024). Coding regions, however, contain a significantly lower proportion of Neandertal-shared variants than introgressed variants (1.2% versus 2.1%, *p* = 0.02; FDR adjusted *p* = 0.04) while the comparison to modern human specific indels shows a non-significant trend in the opposite direction (3.0% versus 2.0%, *p* = 0.05; FDR adjusted *p* = 0.10). These results raise the possibility that introgressed indels have been subjected to stronger negative selection, either before or after the introgression event, compared to modern human specific indels.

### Genome wide association studies (GWAS) and Introgressed Indels

To find further evidence for a potential impact of introgressed indels on human phenotypes, we searched for introgressed indels that are in perfect linkage to SNPs that are linked to specific traits by genome wide association studies (Table 3). We found 9 traits (*p* < 1e-5) related to neurological, immunological, developmental and metabolic phenotypes, among others. Interestingly, one SNP at chromosome 2: 157,096,776 (in perfect linkage disequilibrium (LD) with an indel in chromosome 2: 157,099,707) is associated with menarche [36]. Human carriers of the Neandertal allele showed an earlier menarche compared to non-carriers and the Neandertal allele has a higher prevalence in Europeans (allele frequency = 0.06) compared to Asians (allele frequency = 0.01).

**Table 3:** Introgressed indels linked to genome-wide association studies candidates

Chr	Indel pos.	SNP pos.	SNP rs ID	*P*-value	Trait	EAS_AF	EUR_AF	Gene	C-score(indel)	Ref.
1	196,365,712	196,376,474	rs16839886	7.26E-06	Age-related macular degeneration	0.0129	0.0746	KCNT2	8.613	[59]
1	209,987,712	209,988,047	rs10863790	1.00E-14	Cleft lip	0.4286	0.0139	NA	4.657	[60]
1	210,174,981	210,174,417	rs11119388	4.57E-09	Cleft lip	0.4454	0.0089	SYT14	9.739	[60]
14	55,769,446	55,808,151	rs17673930	1.89E-40	Protein biomarker	0.006	0.0805	CHMP4BP1	6.577	[61]
2	157,099,707	157,096,776	rs17188434	1.00E-09	Menarche (age at onset)	0.0099	0.0606	NA	6.499	[36]
3	23,386,162	23,385,942	rs17013049	2.78E-06	Type 2 diabetes	0.1131	0.0239	UBE2E2	6.473	[62]
3	100,671,648	100,647,927	rs13060137	8.96E-08	Suicide attempts in bipolar disorder	0.002	0.1531	RNU6-865P	3.313	[63]
8	20,253,488	20,263,408	rs1016646	9.45E-06	Preeclampsia	0.0923	0.0636	NA	2.605	[59]
9	87,171,753	87,177,586	rs35640669	5.17E-08	Insulin-related traits	0.0546	0.0348	NA	0.207	[64]

*EAS AF* East Asian allele frequency;*EUR AF* European allele frequency

**Fig. 4:**
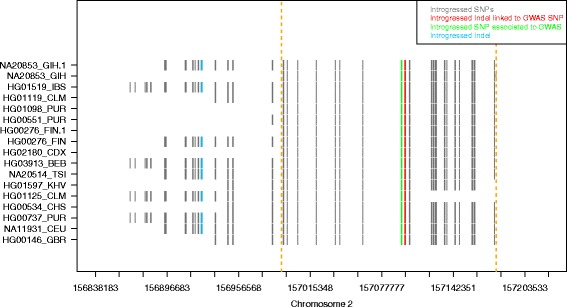
Introgressed region around an introgressed indel linked to menarche. Introgressed haplotypes carrying introgressed indels (*red*) linked to an introgressed SNP associated with menarche GWAS (*green*) in individuals from 1000 Genomes phase 3. The borders of the shared region over all introgressed haplotypes are indicated by the *dashed* *orange* lines

To further corroborate that the menarche associated indel is introgressed, we plotted putatively introgressed variants in the individuals from the 1000 genomes surrounding the location of the indel (Fig. 4). In concordance with the low frequency in present-day Europeans and East-Asians, few individuals showed the homozygous derived state for introgressed variants in the vicinity of the indel. We observe haplotypes of different lengths, two of which encompass an additional introgressed indel upstream. Regions overlapping the indel have also been found to be introgressed in two independent maps of introgressed segments in non-Africans [4, 5].

Considering introgressed variants shared between non-African individuals, we estimate a minimum length of 180,900 bp for the introgressed segment. The recombination rate in this region is 0.23 cM/Mb, which is lower than the genome wide average of ca. 1 cM/Mb [37]. We calculated the probability of a region to retain a length of at least ~180 kb if it was generated by incomplete lineage sorting (see [9, 38]) and found that this scenario is unlikely (*p* = 0.003).

### Gene ontology enrichment

To test whether any group of functionally related genes experienced a shift in constraint from before the split to after the split from Neandertals, we used the Gene Ontology to group and compare the number of shared and modern human specific indels annotated to genes. Two Gene Ontology categories, *ion channel complex* and *transmembrane complex*, showed significant enrichment for modern human specific indels compared to shared indels (Additional file 1: Table S3). This result could be explained by a relaxation of constraint for these genes in modern humans since the split from Neandertals. No significant enrichment was found in the opposite direction, or when comparing introgressed indels to shared indels.

### List of potentially disruptive indels

Identifying the molecular basis for modern human specific traits remains a challenge for the study of human evolution. Here we provide a list of candidates that have been fixed in modern humans since the split from Neandertals and that are annotated as a top 1% disruptive change according to the CADD package (Additional file 1: Table S1). Further study is needed to test whether some of these changes play a role in modern human specific traits.

In addition, we provide a list of putatively introgressed indels which have been classified as likely disruptive (Additional file 1: Table S2). Variants with the highest allele frequency differences (measured by F_ST_) between Europeans and East Asians that also show some evidence for disruptiveness are listed in Additional file 1: Table S4.

## Discussion

Small indels are a common type of sequence variation among present-day humans [39]. Here we used several outgroups to divide indels into derived insertions and derived deletions. Each class was further categorized using the Neandertal genome into those derived variants that are shared with Neandertals and those that are only observed in modern humans.

Previous studies have compared allele frequencies and the proportion of fixed to polymorphic insertions and deletions to gain insight into differences in selection pressures affecting each type of change. Some of these studies found that deletions appear to be more deleterious than insertions [21] while others found the opposite [22], a discrepancy that may in parts be explained by homoplasy, i.e. the independent formation of identical indels on several lineages (Additional file 1: Table S7) [19]. Here we used seven primate outgroups to reduce the effect of homoplasy and to confidently call the ancestral state. Comparing allele frequencies, fixed to polymorphic indels, and Neandertal-shared indels to modern human specific, we found that the proportion of deletions is consistently smaller for older time-frames and higher frequencies, suggesting that deletions are on average more deleterious than insertions. Interestingly, this signal is further corroborated by the genomic distribution of insertions and deletions, where we found a higher fraction of insertions in coding regions compared to deletions, which show a higher fraction that fall in intergenic regions. Despite these consistent results, we caution that our strong requirement of several primate outgroups selects for sites that remain stable over millions of years of evolution, and that our results only hold for this subset of indels, which will be biased towards conserved and against repetitive genomic regions. We also caution that insertions and deletions are influenced by other factors than selection [31–33], and that they may form at unequal rates in different functional classes of the genome.

In principle, a Neandertal-shared derived variant could originate through two processes: either the variant came into existence before the Neandertal and modern human populations split, or the variant was contributed to modern humans after the split, through admixture. We make use of previous results that found Neandertal admixture in out-of-African populations to select indels that likely entered through admixture by selecting those Neandertal-shared variants that are only observed in out-of-African populations. Putatively introgressed indels showed similar differences in the genome-wide distribution of insertions and deletions, with a higher fraction of insertions residing in coding regions and a higher fraction of deletions in intergenic regions. This suggests that introgressed deletions are more deleterious than introgressed insertions.

At least 40% of the introgressing Neandertal genomes can be reconstructed from Neandertal segments segregating in out-of-African populations [4, 5]. However, the distribution of these segments has been found to be non-uniform, with genes and conserved regions of the genome showing an underrepresentation of Neandertal introgression. The patterns of depletion of Neandertal-ancestry near genes have been used to estimate the strength of selection against introgressed segments [7] and simulations suggest that Neandertals may have had a reduction in fitness compared to modern humans [6]. Comparing Neandertal-shared indels, which represent older events and which are mostly fixed, to putatively introgressed indels, we find no evidence for stronger negative selection acting on introgressed variants. However, compared to derived indels on the modern human lineage, Neandertal introgressed variants show some signals that are compatible with more selective constraint, suggesting that selection acted on these variants either before or after introgression.

Some introgressed indels may also convey an advantage to the carrier and there are several examples of variants that have been positively selected after introgression [9, 10, 12, 13]. Among the introgressed indels that were present in both Europeans and East-Asians and that scored highest for affecting phenotype we found a frame shift insertion in PTCHD3 (patched domain-containing protein-3), a gene which has a role in sperm development or sperm function [40] and that has been found to contain a risk-allele for asthma [41]. However, due to the high-frequency in which null-mutations are encountered in present-day humans, the gene has also been suggested to be non-essential in humans [42]. Some introgressed indels were also in perfect linkage with SNPs associated with different traits and diseases in genome-wide association studies. One such indel was linked to a variant associated with a decrease in the time to menarche in humans. The direction of effect for this variant is in line with research suggesting that Neandertals may have reached adulthood earlier than present-day humans [43, 44].

## Conclusions

Indels in modern humans contribute not only to genetic variation, but also appear to be subject to stronger selective forces than nucleotide substitutions. Here, we studied the differences between insertions and deletions using the Neandertal genome as an additional outgroup and found signals that suggest that deletions are more often deleterious than insertions. Among the indels segregating in modern humans are those that entered out-of-African populations by admixture with Neandertals. While these introgressed indels show weak signals of negative selection compared to other variants that segregate in modern humans, we find some variants that may contribute to functional variation in present-day humans. Arguably the most interesting variant with phenotype association is an introgressed indel variant associated with a decreased time to menarche, raising the possibility that some of the introgressing Neandertals’ life history traits now form part of the modern human variation.

## Methods

### Primate multiple sequence alignment

Pairwise alignments between the human reference genome (Lander, Linton et al. 2001) (GhRch37/hg19) and six primates (chimpanzee [45] (panTro4), gorilla [46] (gorGor3), orangutan [47] (ponAbe2), gibbon [48] (nomLeu1), rhesus macaque [49] (rheMac3) and marmoset [50] (calJac3)) were downloaded from the UCSC genome browser [51] and converted into MAF format. In addition, the bonobo [52] (panpan1.1) pairwise whole genome alignment to hg19 was prepared in house following the processing applied to genomes for inclusion in the UCSC genome browser. All seven pairwise alignments were joined into one multiple sequence alignment using the reference guided alignment program multiz (Version: roast.v3; Command-line: “roast + E=hg19 '(((((hg19(panTro4,panpan1.1) gorGor3)ponAbe2)nomLeu1)rheMac3)calJac3)' <input_files.sing.maf> <output_file.maf>” , [53]). The resulting file was filtered to retain only those alignment blocks that include sequence from the genomes of all eight species.

### Inferring fixed derived and polymorphic indels on the human lineage

Human polymorphic indels were extracted from the 1000 Genomes phase 3 dataset [54]. The indels were further filtered by requiring overlap with the eight species whole genome alignment and requiring all seven non-human reference sequences in this alignment to agree. The ancestral state of polymorphic indels was then called as the non-human state and the alternative labeled as a derived human-specific indel. Further filtering was carried out to remove sites with more than one derived variant and long variants marked as variable in copy number (denoted as <CN> for the derived state in the 1000 Genomes data).

Human-specific derived indels were called fixed if all non-human species showed an identical insertion or deletion difference compared to the human reference sequence and if the position was not listed as polymorphic in the 1000 Genomes data.

### Inferring modern human specific indels and putatively introgressed indels using the Neandertal genome

We used the genotype calls of a Neandertal from the Altai Mountains [3] to divide derived human-specific indels into those that are shared with Neandertals and those that are specific to modern humans.

Two percent of the genomes of present day non-Africans show high similarity to the Neandertal genome due to a recent admixture event with Neandertals [3]. To infer putatively introgressed indels we used our set of human polymorphic indels and filtered for variants that are fixed in individuals from sub-Saharan African populations (Luhya, Yoruba, Gambian, Mende and Esan) and show an alternate allele in the Europeans (Utah, Finland, British and Scotland, Iberian, Toscani) or East-Asians (Chinese Dai, Han Chinese, Southern Han Chinese, Japanese, Kinh) that is shared with the Neandertal. We used the same process to infer introgressed SNPs.

### Contrasting fixed and polymorphic insertions and deletions

The McDonald–Kreitman test [27] compares the number of polymorphic changes within one species to the number of fixed changes when comparing to another species between two types of sites, neutral and non-neutral. Under neutrality the ratio of non-neutral to neutral changes is expected to be equal when comparing fixed to polymorphic changes. Negative selection is expected to reduce the number of non-neutral changes that reach fixation, while repeated positive selection is expected to increase the number of non-neutral changes due to the rapid fixation of advantageous alleles. Following the approach of Sjödin et al. and Kvikstad and Duret [19, 21], we applied the concept of the McDonald-Kreitman test to indels by comparing the number of insertions and deletions that are polymorphic to those that are fixed-derived on the human-lineage. *P*-values were calculated using Fisher’s exact test as implemented in R [54].

### Derived site frequency spectra of polymorphic indels

We used the average allele-frequency for different populations from the 1000 Genomes phase 3 data to tabulate the site frequency spectra. Site frequency spectra were compared by applying a two-sided Wilcoxon rank sum test with continuity correction to the distribution of indel frequencies.

The minor allele frequencies for potentially introgressed indels in the European populations and the East Asian populations from the 1000 Genomes Project phase 3 were tabulated to arrive at an AFS of introgressed indels.

### Annotation of indels

Indels were annotated using the variant effect predictor (VEP) [30] version 78 using the option “–most_severe” to limit the output to one annotation per indel. For each annotated region and for each pair of classes of indels, we determined the significance by calculating Fisher’s exact test on a 2 × 2 contingency table contrasting the two classes and the counts inside and outside of the annotated region. The combined list of *p*-values from all variance effect predictor tests was FDR adjusted using the p.adjust() function implemented in R.

In addition the Combined Annotation Dependent Depletion (CADD v1.3) tool [55] was used to score the tentative phenotypic impact of indels. CADD annotates each indel with a phred-scaled C-score. A cutoff of 20 on the C-score was applied to generate lists of indels with an increased chance of affecting phenotype.

### Genome wide association studies

We used a collection of genome-wide association studies (GWASdb, version: 2015 August, hg19 dbSNP142, [56]) to find potential phenotype associations for introgressed indels. Since indels are typically excluded in the process of GWAS, we sought to detect SNPs that are in perfect LD with introgressed indels in the 1000 Genomes. Indels that showed an identical combination of reference/non-reference genotypes as the GWAS associated SNP in all individuals were considered completely linked. We report phenotype associations for each indel that is in perfect LD with a SNP that has been associated with the corresponding phenotype with a *p*-value of at least 1e-5.

### Gene ontology enrichment

Enrichment of indels in specific gene categories was tested using the software package FUNC version 0.4.7 [57]. For this, we selected indels that were assigned to genes based on the VEP annotation and further annotated these indels to gene categories used the Gene Ontology. To account for all the plausible effects, for instance when an indel overlaps more than one gene, we allowed multiple annotations of each indel. Genes were assigned corresponding GO categories using the Ensembl database [version: Ensembl Genes 75 (GRCh37)] [58].

In addition to explanations involving selection, the number of indels in a gene category can vary due to differences in mutation rates or due to a difference in gene-length between categories. In order to avoid these issues, we compared the number of two types of indels per category using the FUNC implementation of the binomial test. The following types of indels were compared:

1. Indels shared with Neandertals to those that are modern human specific
2. Indels that are shared with Neandertals to those that introgressed from Neandertals.

We chose a *p*-value cutoff of less than or equal to 0.05 for the family wise error rate (FWER) to filter for significantly enriched categories.

## Additional file

Additional file 1:
**Tables S1-S7.** and **Figures S1-S4.** Full legends are contained within the file. (PDF 378 kb)

## Abbreviations

AFS: Allele Frequency Spectrum
FDR: False Discovery Rate
Indel: Insertion or Deletion
LD: Linkage Disequilibrium
SNP: Single Nucleotide Polymorphism
