# Supplementary material for: Using the Neandertal genome to study the evolution of small insertions and deletions in modern humans
Source: BMC Evol Biol. 2017 Aug 4;17:179. doi: 10.1186/s12862-017-1018-8 (PMC5543596; doi:10.1186/s12862-017-1018-8)
Supplement: Additional file 1: — Tables S1-S7. and Figures S1-S4. Full legends are contained within the file. (PDF 378 kb) [file 12862_2017_1018_MOESM1_ESM.pdf]

Using the Neandertal genome to study the evolution of small insertions and deletions in modern humans

Manjusha Chintalapati, Michael Dannemann, Kay Prüfer

Additional File 1 with Tables S1-S7 and Figures S1-S4.

Chr	Position	Ref	alt	Type	Consequence	Gene	c-score
7	115542344	TAGAG	T	Del	Intergenic	NA	22.1
3	25739437	C	CA	Ins	Intergenic	NA	22.1
2	221679644	TAATC	T	Del	Intergenic	NA	21.9
7	156283580	CA	C	Del	Intronic	LINC01006	21.4
2	160083677	AGAGT	A	Del	Intronic	TANC1	21.3
9	119310385	CTGTT	C	Del	Intronic	RP11-264C15.2	21.1
9	119310385	CTGTT	C	Del	Intronic	ASTN2	21.1
9	37265129	C	CT	Ins	Intronic	ZCCHC7	21.0
8	65910625	ATAGT	A	Del	Intergenic	NA	20.7
12	122590799	TTC	T	Del	Intronic	MLXIP	20.6
2	168891430	C	CA	Ins	Intronic	STK39	20.2
2	144225349	CTT	C	Del	Intronic	RP11-570L15.2	20.2
2	144225349	CTT	C	Del	Intronic	ARHGAP15	20.2
2	144225349	CTT	C	Del	Intronic	AC096558.1	20.2
20	40295358	CA	C	Del	Intergenic	NA	20.2
20	38267544	GC	G	Del	Intergenic	NA	20.2
11	117229118	AAT	A	Del	Intronic	CEP164	20.1
1	108038937	G	GC	Ins	Intergenic	NA	20.1

Table S1: Top 1% c-score fixed modern human indels.

Chr	Positions	Ref	Alt	Type	Consequence	Gene	C-score	EAS_AF	EUR_AF
14	74060511	T	TTCAA	Ins	Frame_shift	ACOT4	34	0	0.006
22	23011159	AG	A	Del	Frame_shift	IGLV3-27	24.8	0.0139	0
6	146185477	T	TA	Ins	Frame_shift	SHPRH	24.3	0.0516	0
2	236693080	CTAAT	C	Del	Upstream	AC064874.1	22.9	0.0149	0
10	27687534	C	CT	Ins	Frame_shift	PTCHD3	22.9	0.2242	0.0119
7	21068784	T	TG	Ins	Intergenic	NA	22.8	0	0.0099
22	24313530	GGA	G	Del	Frame_shift	DDTL	22.7	0.0278	0.002
4	151508852	CA	C	Del	Downstream	MAB21L2	22.6	0	0.004
2	179301055	CAG	C	Del	Intronic	PRKRA	22.6	0.1538	0.0249
2	177503917	C	CT	Ins	Upstream	LINC01116	22.6	0.004	0
2	177503917	C	CT	Ins	Intronic	LINC01117	22.6	0.004	0
16	28915046	C	CTT	Ins	Downstream	RABEP2	22.3	0.0109	0
4	117649011	CT	C	Del	Intergenic	NA	22.2	0	0.0388
13	72876666	CA	C	Del	Intergenic	NA	22.2	0	0.0159
1	209738399	TG	T	Del	Intronic	RP1-272L16.1	22.2	0.245	0.0119
9	98096123	TAA	T	Del	Intergenic	NA	22.1	0.0446	0
3	184071131	C	CCGG	Ins	Inframe	CLCN2	22.1	0	0.0229
14	99742823	TTA	T	Del	Upstream	BCL11B	22.1	0.0139	0
1	205293177	TAAAC	T	Del	Upstream	NUAK2	22.0	0.002	0.008

12	102125452	TATAAA	T	Del	Downstream	CHPT1	21.9	0.2917	0.008
11	44026804	AC	A	Del	Downstream	RP11-613D13.4	21.9	0.0288	0.001
6	69910260	TA	T	Del	Intronic	BAI3	21.8	0	0.001
14	99240729	GT	G	Del	Intergenic	NA	21.8	0.1716	0.0388
14	66722909	ATAAT	A	Del	Intronic	RP11-72M17.1	21.8	0	0.0189
8	4762649	TA	T	Del	Intronic	CSMD1	21.7	0.0437	0
14	65936081	ATAG	A	Del	Upstream	RPL21P8	21.7	0.0873	0.003
8	107927163	AAC	A	Del	Intergenic	NA	21.6	0	0.003
5	175215413	TG	T	Del	Intergenic	NA	21.6	0	0.008
5	58519627	CCAAT	C	Del	Intronic	PDE4D	21.4	0.0536	0.004
5	117827264	TTTAA	T	Del	Intronic	CTD-2281M20.1	21.4	0.0883	0.003
18	75697997	CA	C	Del	Upstream	LINC01029	21.4	0.0625	0.0249
1	46966402	TA	T	Del	Intergenic	NA	21.4	0.0536	0
1	14020411	C	CAG	Ins	Downstream	SCARNA11	21.4	0.0218	0.0209
11	94667080	CAAG	C	Del	Intergenic	NA	21.3	0	0.0149
5	52608156	C	CT	Ins	Intergenic	NA	21.2	0.0645	0
1	83216710	TTAAG	T	Del	Intergenic	NA	21.2	0.0308	0
17	37815323	TGAA	T	Del	Inframe	STARD3	21.2	0	0.003
1	218868860	AGTTT	A	Del	Intergenic	NA	21.2	0.005	0.0666
2	223154225	T	TG	Ins	Intronic	PAX3	21.1	0.001	0.001
16	79076425	TACTC	T	Del	Intronic	WWOX	21.1	0.001	0.0497
5	154878448	CAAT	C	Del	Intergenic	NA	21.0	0.0466	0
3	169381200	C	CT	Ins	Regulatory	NA	21.0	0.0774	0.002

Table S2: Top 1% c-score introgressed indels

Component	Category	Gene ontology	Genes in Modern human specific	Genes in Shared with Neandertals	FWER_ Modern_ human	FWER_ Shared
cellular_component	ion channel complex	GO:0034702	15748	7377	0.005	1
cellular_component	transmembrane transporter complex	GO:1902495	16123	7559	0.005	1

Table S3: Gene Ontology categories with enrichment for modern human specific changes.

Chr	Position	Ancestral	Derived	Type	Annotation	Gene	C-score	F_{st}	EAS_AF	EUR_AF
11	120175419	TAGAAA	T	Del	Regulatory	NA	17.71	0.599	0.6032	0.002
12	102374341	T	TAG	Ins	Intronic	DRAM1	11.54	0.42404	0.4554	0.0149
1	209986054	GTGAC	G	Del	Intergenic	NA	10.82	0.39851	0.4286	0.0139
1	215924632	CAGT	C	Del	Intronic	USH2A	11.57	0.3787	0.3968	0.008
14	58320402	TA	T	Del	Intronic	SLC35F4	16.76	0.36089	0.3919	0.0139
7	13620958	CT	C	Del	Intronic	AC011288.2	12.06	0.34519	0.001	0.3479
12	114745134	TA	T	Del	Intergenic	NA	12.74	0.30133	0.3482	0.0209
3	169313681	GA	G	Del	Intronic	MECOM	10.53	0.28547	0.2887	0.001
2	169866296	ACT	A	Del	Intronic	ABCB11	15.80	0.2778	0.2788	0
12	102125452	TATAAA	T	Del	Downstream	CHPT1	21.9	0.272	0.2917	0.008
12	102622272	CT	C	Del	Downstream	RP11-18O15.1	10.18	0.26756	0.3006	0.0139
16	72814910	G	GT	Ins	Downstream	ZFHX3	12.19	0.25482	0.2679	0.005
6	131324263	TA	T	Del	Intronic	EPB41L2	13.38	0.25264	0.2609	0.003
10	27845466	CAG	C	Del	Intergenic	NA	10.66	0.23812	0.2629	0.0099
6	131069041	AAAG	A	Del	Intergenic	NA	14.48	0.23294	0.2688	0.0149
5	36203135	AAGAG	A	Del	Regulatory	NA	15.35	0.22423	0.2252	0
5	36194785	TTCTC	T	Del	3prime_utr	NADK2	11.07	0.22423	0.2252	0
5	36193223	CAG	C	Del	Downstream	NADK2	20.3	0.22423	0.2252	0
1	209738399	TG	T	Del	Intronic	RP1-272L16.1	22.2	0.21545	0.245	0.0119
12	125147092	ATGGCC	A	Del	Intergenic	NA	10.11	0.20072	0.3145	0.0547

Table S4: Introgressed indels with F_{st} between Europeans and East Asians above 0.15 and c-score above 10.

Category	Utr	Downstream	Coding_Region	Intergenic	Intron	Non_Coding_Transcript_Exon	Regulatory_Region	Splice	Upstream
Shared	2.3	3.4	0.118	31.189	55.68	1.497	1.739	0.1912	3.86
Modern	2.61	3.5	0.309	30.324	55.89	1.59	1.75	0.2007	3.849
Introgressed	2.46	3.4	0.209	31.51	55.3	1.57	1.55	0.176	3.818

Table S5: Percentage of indels annotated using VEP for Neandertal-shared indels, modern human specific indels and introgressed indels.

Category	Shared	Modern Human specific
Deletions	199041	604423
Insertions	152840	266969
Ratio(Deletions/Insertions)	1.30	2.26

Table S6A: Counts of insertions to deletions compared between modern human specific and Neandertal shared indels. Introgressed indels were removed from the counts of Neandertal-shared indels. The ratios differ significantly (Fisher's exact test p<2.2e-16, odds ratio=0.58)

	Before filtering introgressed indels from Neandertal-shared			After Filtering introgressed indels from Neandertal-shared		
Category	p-value	Odds ratio	FDR	P-value	Odds ratio	FDR
Intergenic	1.77e-21	0.9599	9.57e-21	3.01e-21	0.96020	3.22e-20
Intronic	0.0369	1.0087	0.083	0.0233	1.008044	0.100849

Table S6B: Proportion of Neandertal-shared vs modern human specific indels in intergenic and intronic regions before and after filtering introgressed indels.

Human Allele	Non-Human Allele	count
**7 primates differ**		
Hg19	chimpanzee, bonobo, gorilla, orangutan, gibbon, rhesus, marmoset	336,559
**6 primates differ**		
Hg19, gorilla	chimpanzee, bonobo, orangutan, gibbon, rhesus, marmoset	12932
Hg19, marmoset	chimpanzee ,bonobo, gorilla, orangutan, gibbon, rhesus	9797
Hg19, rhesus	chimpanzee, bonobo, gorilla, orangutan, gibbon, marmoset	5253
Hg19, gibbon	chimpanzee, bonobo, gorilla, orangutan, rhesus, marmoset	4017
Hg19, orangutan	chimpanzee, bonobo, gorilla, gibbon, rhesus, marmoset	3829
Hg19, bonobo	chimpanzee, gorilla, orangutan, gibbon, rhesus, marmoset	3292
Hg19, chimpanzee	bonobo, gorilla, orangutan, gibbon, rhesus, marmoset	3710
**5 primates differ**		
Hg19, chimpanzee, bonobo	gorilla, orangutan, gibbon, rhesus, marmoset	84442
Hg19, rhesus, marmoset	chimpanzee, bonobo, gorilla, orangutan, gibbon	2100
Hg19, chimpanzee, gorilla	bonobo, orangutan, gibbon, rhesus, marmoset	2048
Hg19, gorilla, marmoset	chimpanzee, bonobo, orangutan, gibbon, rhesus	1117
Hg19, gorilla, orangutan	chimpanzee, bonobo, gibbon, rhesus, marmoset	1115
Hg19, gibbon, marmoset	chimpanzee, bonobo, gorilla, orangutan, rhesus	975
Hg19, bonobo, gorilla	chimpanzee, orangutan, gibbon, rhesus, marmoset	953

Hg19, gorilla, gibbon	chimpanzee, bonobo, orangutan, rhesus, marmoset	835
Hg19, orangutan, marmoset	chimpanzee, bonobo, gorilla, gibbon, rhesus	802
Hg19, orangutan, gibbon	chimpanzee, bonobo, gorilla, rhesus, marmoset	795
Hg19, gibbon, rhesus	chimpanzee, bonobo, gorilla, orangutan, marmoset	727
Hg19, gorilla, rhesus	chimpanzee, bonobo, orangutan, gibbon, marmoset	705
Hg19, orangutan, rhesus	chimpanzee, bonobo, gorilla, gibbon, marmoset	634
Hg19, chimpanzee, marmoset	bonobo, gorilla, orangutan, gibbon, rhesus	352
Hg19, bonobo, marmoset	chimpanzee, gorilla, orangutan, gibbon, rhesus	349
Hg19, chimpanzee, gibbon	bonobo, gorilla, orangutan, rhesus, marmoset	285
Hg19, chimpanzee, orangutan	bonobo, gorilla, gibbon, rhesus, marmoset	279
Hg19, chimpanzee, rhesus	bonobo, gorilla, orangutan, gibbon, marmoset	219
Hg19, bonobo, rhesus	chimpanzee, gorilla, orangutan, gibbon, marmoset	206
Hg19, bonobo, gibbon	chimpanzee, gorilla, orangutan, rhesus, marmoset	199
Hg19, bonobo, orangutan	chimpanzee, gorilla, gibbon, rhesus, marmoset	178
**4 primates differ**		
Hg19, chimpanzee, bonobo, gorilla	orangutan, gibbon, rhesus, marmoset	371776
Hg19, gorilla, orangutan, gibbon	chimpanzee, bonobo, rhesus, marmoset	1952
Hg19, gibbon, rhesus, marmoset	chimpanzee, bonobo, gorilla, orangutan	1123
Hg19, orangutan, gibbon, rhesus	chimpanzee, bonobo, gorilla, marmoset	1085
Hg19, chimpanzee, gorilla, orangutan	bonobo, gibbon, rhesus, marmoset	869
Hg19, orangutan, rhesus, marmoset	chimpanzee, bonobo, gorilla, gibbon	818
Hg19, gorilla, rhesus, marmoset	chimpanzee, bonobo, orangutan, gibbon	771
Hg19, gorilla, gibbon, rhesus	chimpanzee, bonobo, orangutan, marmoset	744
Hg19, bonobo, gorilla, orangutan	chimpanzee, gibbon, rhesus, marmoset	270
Hg19, chimpanzee, orangutan, gibbon	bonobo, gorilla, rhesus, marmoset	227
Hg19, bonobo, rhesus, marmoset	chimpanzee, gorilla, orangutan, gibbon	218
Hg19, chimpanzee, rhesus, marmoset	bonobo, gorilla, orangutan, rhesus	198
Hg19, chimpanzee, gibbon, rhesus	bonobo, gorilla, orangutan, marmoset	121
Hg19, bonobo, orangutan, gibbon	chimpanzee, gorilla, rhesus, marmoset	95
Hg19, bonobo, gibbon, rhesus	chimpanzee, gorilla, orangutan, marmoset	93
**3 primates differ**		
Hg19, chimpanzee, bonobo, gorilla, orangutan	gibbon, rhesus, marmoset	133428
Hg19, orangutan, gibbon, rhesus, marmoset	chimpanzee, bonobo, gorilla	12983
Hg19, gorilla, orangutan, gibbon, rhesus	chimpanzee, bonobo, marmoset	8204
Hg19, gorilla, gibbon, rhesus, marmoset	chimpanzee, bonobo, orangutan	2891
Hg19, chimpanzee, gorilla, orangutan, gibbon	bonobo, rhesus, marmoset	2284
Hg19, bonobo, gorilla, orangutan, gibbon	chimpanzee, rhesus, marmoset	730
Hg19, chimpanzee, orangutan, gibbon, rhesus	bonobo, gorilla, marmoset	373
Hg19, chimpanzee, gibbon, rhesus, marmoset	bonobo, gorilla, orangutan	282
Hg19, bonobo, orangutan, gibbon, rhesus	chimpanzee, gorilla, marmoset	236
Hg19, bonobo, gibbon, rhesus, marmoset	chimpanzee, gorilla, orangutan	204
**2 primates differ**		
Hg19, chimpanzee, bonobo, gorilla, orangutan, gibbon	rhesus, marmoset	559086
Hg19, gorilla, orangutan, gibbon, rhesus, marmoset	chimpanzee, bonobo	219505
Hg19, chimpanzee, bonobo, gorilla, orangutan, rhesus	gibbon, marmoset	42798
Hg19, chimpanzee, bonobo, gorilla, orangutan, marmoset	gibbon, rhesus	36744
Hg19, chimpanzee, bonobo, gorilla, rhesus, marmoset	orangutan, gibbon	35622
Hg19, chimpanzee, bonobo, gorilla, gibbon, rhesus	orangutan, marmoset	30163
Hg19, chimpanzee, bonobo, gorilla, gibbon, marmoset	orangutan, rhesus	21136
Hg19, chimpanzee, orangutan, gibbon, rhesus, marmoset	bonobo, gorilla	12983

Hg19, chimpanzee, gorilla, orangutan, gibbon, rhesus	bonobo, marmoset	8204
Hg19, chimpanzee, bonobo, gibbon, rhesus, marmoset	gorilla, orangutan	5934
Hg19, chimpanzee, bonobo, orangutan, gibbon, rhesus	gorilla, marmoset	5689
Hg19, bonobo, gorilla, orangutan, gibbon, rhesus	chimpanzee, marmoset	4872
Hg19, chimpanzee, gorilla, orangutan, gibbon, marmoset	bonobo, rhesus	4137
Hg19, chimpanzee, gorilla, orangutan, rhesus, marmoset	bonobo, gibbon	3043
Hg19, chimpanzee, gorilla, gibbon, rhesus, marmoset	bonobo, orangutan	2761
Hg19, chimpanzee, bonobo, orangutan, gibbon, marmoset	gorilla, rhesus	2430
Hg19, bonobo, gorilla, orangutan, gibbon, marmoset	chimpanzee, rhesus	2082
Hg19, chimpanzee, bonobo, orangutan, rhesus, marmoset	gorilla, gibbon	1989
Hg19, bonobo, orangutan, gibbon, rhesus, marmoset	chimpanzee, gorilla	1981
Hg19, bonobo, gorilla, orangutan, rhesus, marmoset	chimpanzee, gibbon	1634
Hg19, bonobo, gorilla, gibbon, rhesus, marmoset	chimpanzee, orangutan	1623
**1 primate differs**		
Hg19, chimpanzee, bonobo, gorilla, orangutan, gibbon, rhesus	marmoset	7370356
Hg19, chimpanzee, bonobo, gorilla, orangutan, gibbon, marmoset	rhesus	2637782
Hg19, chimpanzee, bonobo, gorilla, orangutan, gibbon, marmoset	gibbon	1437746
Hg19, chimpanzee, bonobo, gorilla, gibbon, rhesus, marmoset	orangutan	1656095
Hg19, chimpanzee, bonobo, orangutan, gibbon, rhesus, marmoset	gorilla	501071
Hg19, chimpanzee, gorilla, orangutan, gibbon, rhesus, marmoset	bonobo	202408
Hg19, bonobo, gorilla, orangutan, gibbon, rhesus, marmoset	chimpanzee	170578

Table S7: Counts of indels where some or all primate outgroups carry an identical non-human allele

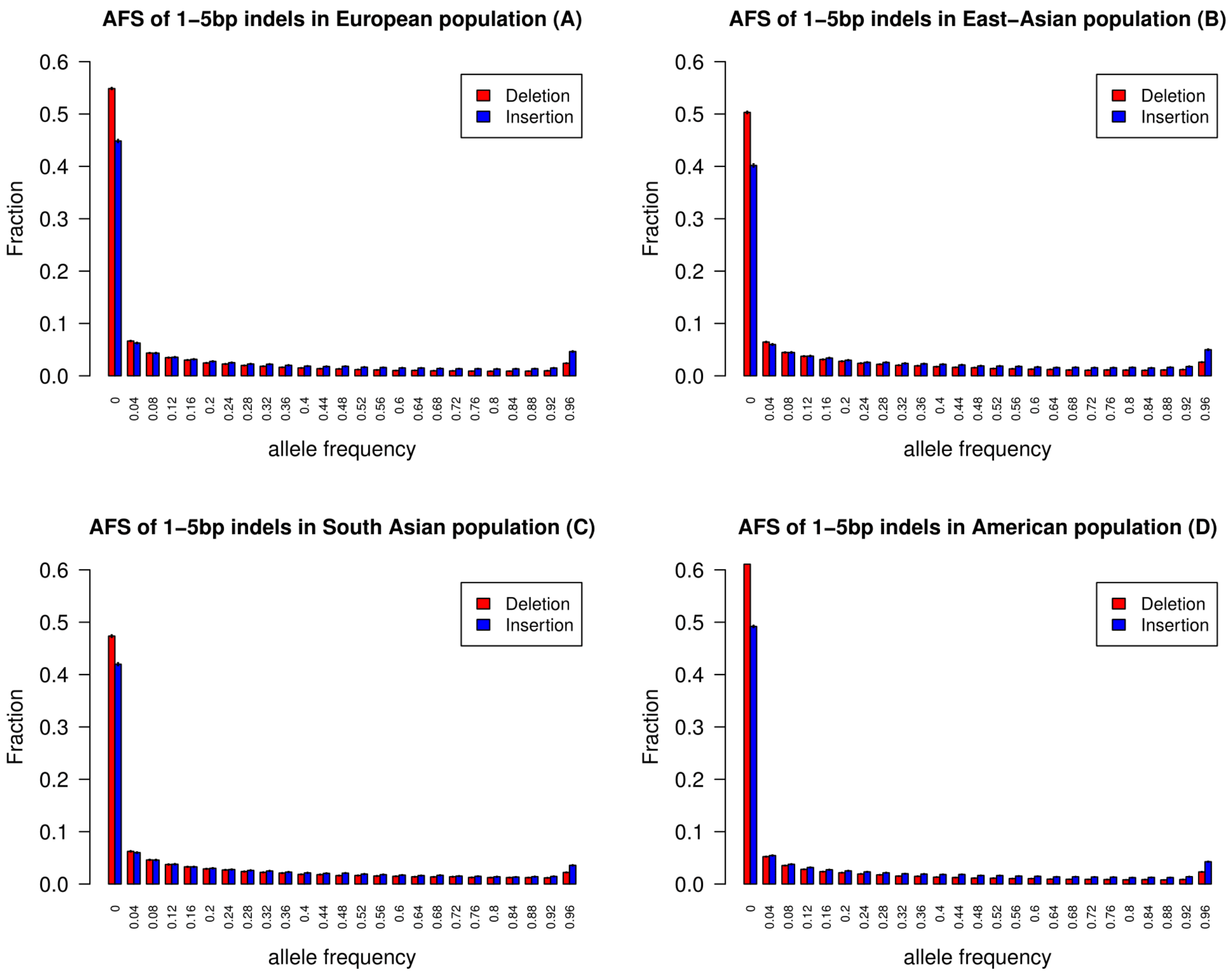

Figure S1: Site frequency spectra (SFS) of human lineage indels in non-African populations: A) European population B) East-Asian population C) South Asian population D) American population for different lengths of indels (1-5bp). All frequency distributions show significant differences (Wilcoxon rank sum test, two-sided: all p-values <2.3e-16; FDR-adjusted p-values<2.2e-16).

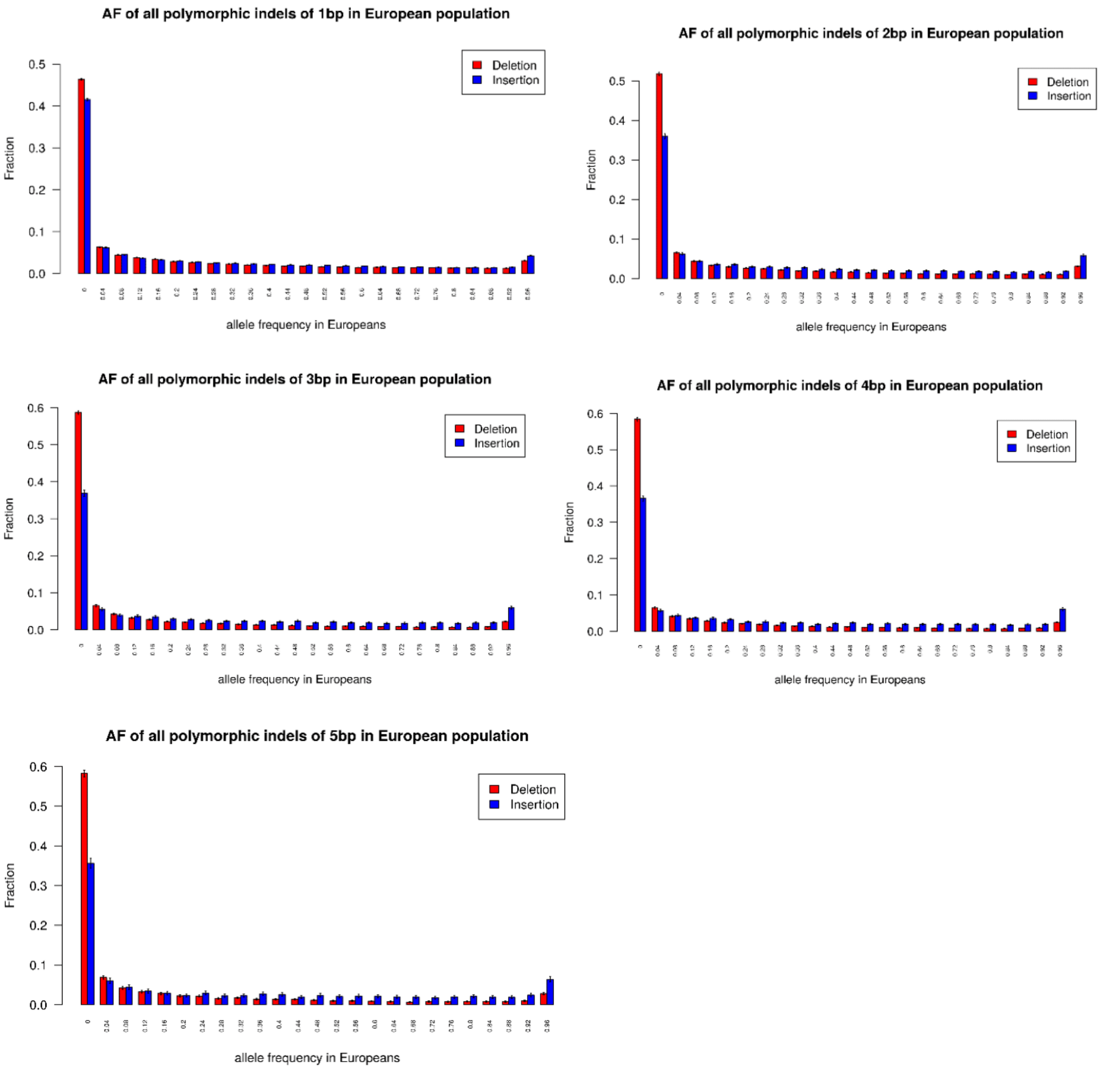

Figure S2: Site frequency spectra (SFS) of human lineage indels in the European population for different lengths of indels (1-5bp). All frequency distributions show significant differences (Wilcoxon rank sum test, two-sided: all p-values <2.3e-16; FDR-adjusted p-values<2.2e-16).

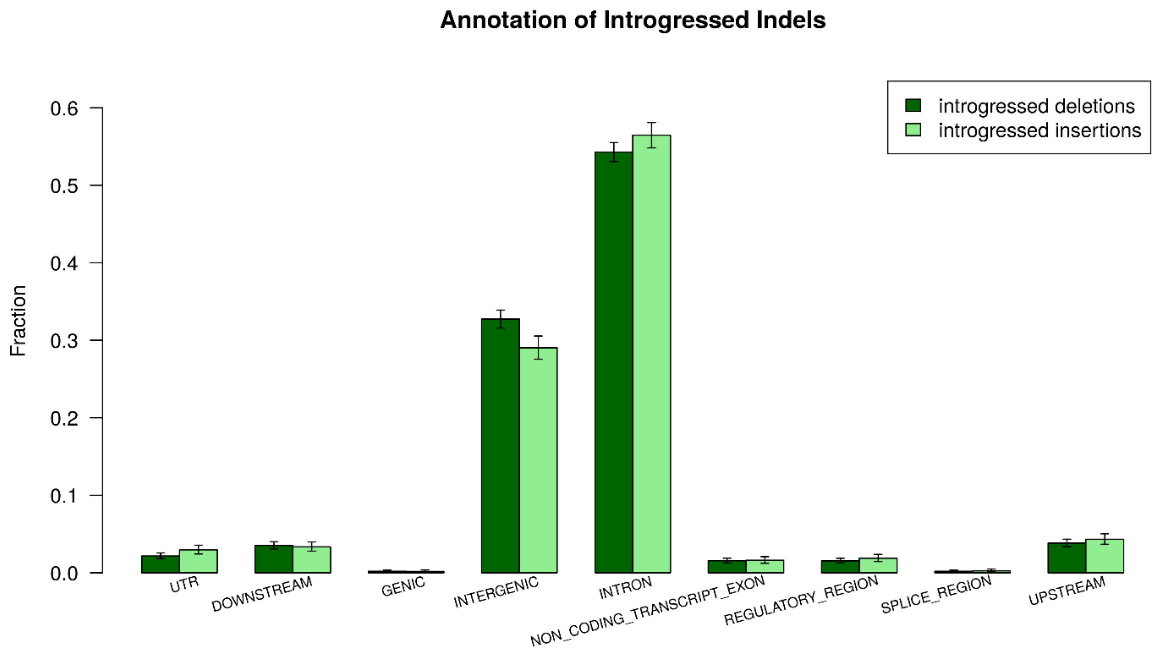

Figure S3: Relative frequency of variant effect predictor annotation of introgressed deletions (dark green) and introgressed insertions (light green).

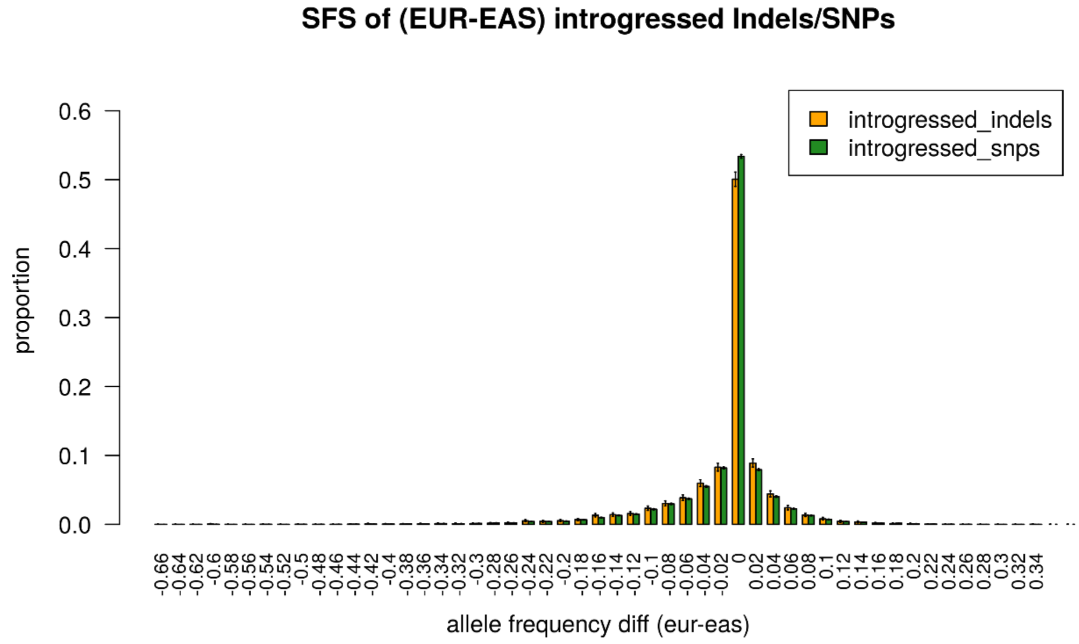

Figure S4: Histogram comparing the European to East-Asian allele frequency differences between indels and SNPs.
